# Fanconi syndrome following administration of oral supplements containing red yeast rice: several months follow-up of three cases

**DOI:** 10.1007/s13730-024-00955-2

**Published:** 2024-12-11

**Authors:** Reina Matsui-Hosoya, Koji Sato, Motohiro Yagasaki, Hitomi Hirose, Yusuke Fukao, Toshiki Kano, Hiroaki Io, Yusuke Suzuki

**Affiliations:** 1 Department of Nephrology, Juntendo University Nerima Hospital, 3-1-10 Takanodai, Nerima-ku, Tokyo, 177-8521 Japan; 2 Department of Nephrology, Faculty of Medicine, Juntendo University, 3-1-3 Hongou, Bunkyo-ku, Tokyo, 133-8431 Japan

**Keywords:** Fanconi syndrome, Acute kidney injury, Tubulointerstitial, Benikoji CholesteHelp^®^, Dietary supplement

## Abstract

To date, the treatment strategy and long-term prognosis of acute kidney injury (AKI) after taking Benikoji CholesteHelp^®^, a red yeast rice supplement, remains unclear. We present three cases wherein renal dysfunction improved within a few months of supplement discontinuation, without immunosuppressive therapy. Case 1: A 59-year-old woman with a history of hypertension, treated with telmisartan (serum creatinine [sCr]: 0.65 mg/dL; estimated glomerular filtration rate [eGFR]: 71.3 mL/min/1.73 m^2^) and Benikoji CholesteHelp^®^ for 7 months, developed Fanconi syndrome (FS) and severe renal impairment (sCr: 2.32 mg/dL; eGFR: 17.7 mL/min/1.73 m^2^). Renal biopsy and gallium-67 scintigraphy revealed no active drug-induced interstitial nephritis. Her condition improved significantly after supplement discontinuation. Her renal function gradually improved, with 3-month follow-up sCr and eGFR values of 0.96 mg/dL and 46.5 mL/min/1.73 m^2^, respectively; however, these were still worse than the pre-onset values. Case 2: A 48-year-old man had mild renal dysfunction (sCr: 1.12 mg/dL; eGFR: 56.76 mL/min/1.73 m^2^) after taking Benikoji CholesteHelp^®^ for approximately 2 years; this was reversed upon supplement discontinuation. Case 3: A 47-year-old man with FS and mild renal dysfunction (sCr: 1.09 mg/dL; eGFR: 58.5 mL/min/1.73 m^2^) after taking Benikoji CholesteHelp^®^ for approximately 4 months, showed notable improvement in FS after supplement discontinuation; however, the mild renal dysfunction persisted. The primary intervention in all cases was immediate supplement discontinuation, leading to rapid improvement in renal function, without need for immunosuppressive therapy. These findings increase our understanding of renal impairment caused by red yeast rice, with improvement after withdrawal, sometimes after several months.

## Introduction

Drug-induced kidney injury is one of the leading causes of acute kidney injury (AKI), with a reported incidence of approximately 15–25% among patients hospitalized for AKI [1, 2]. Yokoyama et al. reported that 1.24% of 26,545 cases registered in the Japanese Renal Biopsy Registry System were diagnosed as drug-induced kidney injury [3]. Furthermore, renal damage can be caused not only by pharmaceuticals and prescription drugs [4, 5] but also by dietary supplements or natural herbs [6]. In supplement-induced kidney injury, it is frequently difficult to identify the specific causative agent because supplements contain various ingredients and additives and because patients use multiple supplements and medications. Even when the causative agent is identified, the prognosis is poor if kidney damage reaches an irreversible stage. Patients should be informed that supplements taken for health can sometimes cause adverse events, including kidney damage. Additionally, medical professionals should recognize that oral medications, including supplements, have the potential to cause unexpected side effects and should consider supplement-induced nephropathy when encountering kidney injury of unknown cause.

Recent reports in Japan have highlighted cases of kidney dysfunction associated with Benikoji CholesteHelp^®^, a supplement of red yeast rice, with investigations ongoing into their accidental contamination with puberulic acid and their potential renal effects. Case reports of kidney injury due to Benikoji CholesteHelp^®^ are actively being reported [7–11]. The effects of puberulic acid on renal function remain unclear, despite its antimalarial and cytotoxic activities being well established [12]. Although numerous reports exist on drug-induced FS [5], there have been few reports of FS due to oral supplementation before the kidney dysfunction cases had reported with Benikoji CholesteHelp^®^. The mechanism by which supplements cause kidney damage remains unclear, warranting further studies to elucidate the causative agents and pathophysiological mechanisms. Here, we report three cases of renal dysfunction after administering Benikoji CholesteHelp^®^, with notable improvement upon supplement discontinuation. Reporting multiple cases related to Benikoji CholesteHelp^®^ may help to elucidate new potential causes of drug-induced kidney injury.

## Case report

### Case 1

A 59-year-old woman with a medical history of hypertension and dyslipidemia was admitted for kidney dysfunction and proteinuria. Approximately 10 months prior, a medical checkup revealed high low-density lipoprotein (LDL) cholesterol levels and hypertension, and 8 months prior, the patient was prescribed telmisartan (20 mg daily) by a local doctor. Her serum creatinine (sCr) level was 0.65 mg/dL, and the estimated glomerular filtration rate (eGFR) was 71.6 mL/min/1.73 m^2^. Seven months prior, she started taking 1–3 tablets daily, with defined proper dosage, of Benikoji CholesteHelp^®^ for dyslipidemia. Two months prior, the patient experienced constipation and fell due to fatigue. She was prescribed loxoprofen (60 mg daily for 3 days) for the bruising caused by the fall. One month prior, the patient had worsening fatigue and foamy urine, and 4 days before admission, her urine test using a commercially available test kit was positive for urinary protein. The patient visited a local doctor, and her urine was positive for protein and occult blood. Her sCr level was also found to be elevated at 2.25 mg/dL, with an eGFR of 18.3 mL/min/1.73 m^2^, leading to a referral to our hospital.

**Fig. 1:**
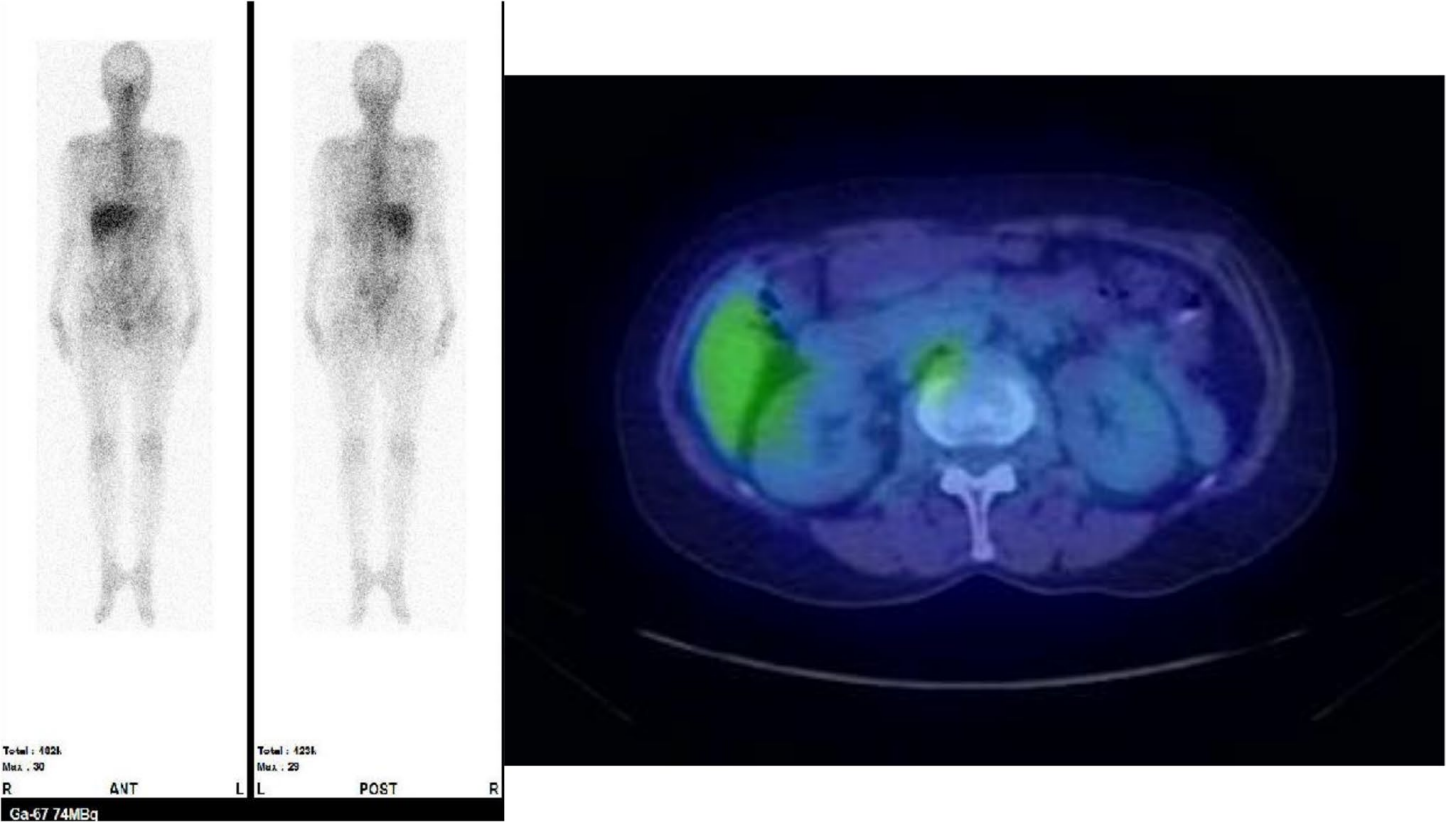
Gallium-67 scintigraphy images (planar coronal image and single-photon emission computed tomography [SPECT] horizontal image). Both images were negative in Case 1

**Fig. 2:**
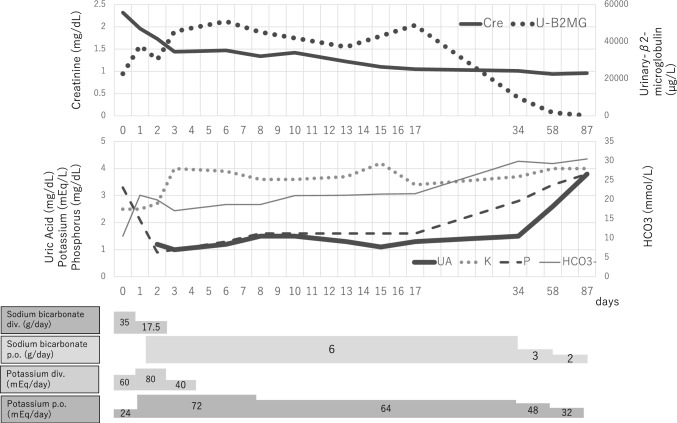
Clinical course after supplement discontinuation in Case 1. The serum levels of creatinine, potassium, phosphorus, and acidosis improved immediately after the discontinuation of the supplement. Serum creatinine and urinary tubular injury markers also recovered

**Fig. 3:**
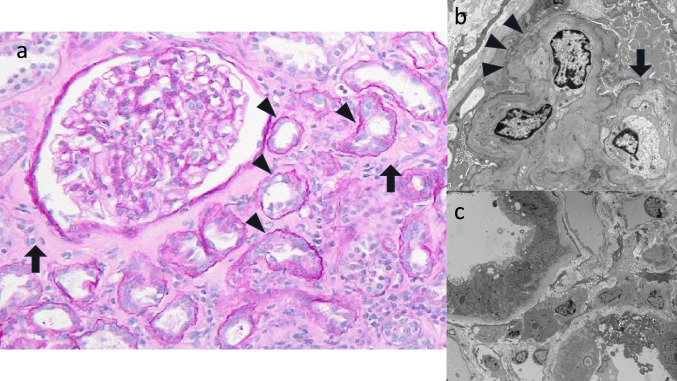
Pathology findings of the kidney in Case 1. Light microscopy (**a**) and electron microscopy (**b**, **c**) findings of the kidney biopsy. Periodic acid Schiff (PAS) stain reveals diffuse interstitial fibrosis (long arrows) with tubular atrophy (short arrows) in 40% of the renal cortex. Glomerular and vascular findings are almost normal (**a**). Morphological abnormalities were not observed in the tubular epithelial cells via electron microscopy (**c**). Some parts of the basement glomerular membrane exhibited wrinkling (short arrows) and duplication (long arrow) (**b**). No abnormality findings were shown on podocyte foot processes

On admission, the patient’s height was 155 cm, weight was 46.2 kg, and vital signs were normal; there was no edema or decreased urinary output. She had general fatigue and limb weakness, with no neurological abnormalities. In addition to renal dysfunction with a sCr level of 2.32 mg/dL and an eGFR of 17.7 mL/min/1.73 m^2^, the patient had hypokalemia (2.5 mmol/L), metabolic acidosis (pH: 7.028, pCO_2_: 30.6 mmol/L, and HCO_3_: 8.0 mmol/L), hyponatremia (130 mEq/L), hypouricemia (1.9 mg/dL), and hypophosphatemia (3.3 mg/dL on admission that decreased to 0.9 mg/dL the day after admission). The urinary pH was at the lower limit of the normal range, and the urine was positive for proteins (3.20 g/gCr); she also had microscopic hematuria (5–9/high-power field), glycosuria, and elevated levels of tubular injury markers (Table 1). The decrease in percentage tubular reabsorption of phosphate (TRP) (61.7%) and increase in fractional excretion of uric acid (FEUA) (45.3%) were attributed to proximal tubular damage, leading to a diagnosis of FS (Table 1). There were no findings suggestive of multiple myeloma, Sjögren’s syndrome, amyloidosis, or malignant tumors (e.g., M protein, autoantibodies, or tumor lesions on imaging) and no history of exposure to possible etiological agents of FS, such as toluene or cadmium. Gallium-67 (Ga) scintigraphy was negative in the kidneys, which is atypical for drug-induced acute interstitial nephritis (Fig. 1). The treatment course during admission is shown in Fig. 2. Considering the possibility of renal injury by medication or supplements, Benikoji CholesteHelp^®^ was discontinued on admission. Telmisartan (20 mg daily) was switched to amlodipine (7.5 mg daily) because of the AKI. Hypokalemia and metabolic acidosis were rapidly corrected within 3 days with intravenous potassium chloride, sodium bicarbonate, and oral medication. Subsequently, the medical replacement dose was gradually reduced and switched from intravenous to oral administration without any worsening in the electrolyte disorder or metabolic acidosis. sCr and urinary tubular injury markers also recovered after supplement discontinuation. To clarify the pathogenic mechanisms in greater detail, renal biopsy was performed on day 14. Tubular atrophy and interstitial fibrosis were observed in approximately 40% of the area, and inflammatory cell infiltration was scattered in some areas. Degeneration was observed in a portion of the remaining proximal tubular epithelial cells, and some of them were shed from the basement membrane. However, on electron microscopy, morphological abnormalities, such as mitochondrial deformity or swelling in the tubular epithelial cells, were rare, suggesting that kidney specimens sampled for electron microscopy were the areas where the injury was relatively mild. Foot process effacement was also absent, despite the presence of proteinuria, which peaked at 3.20 g/gCr. A few glomeruli and blood vessel abnormalities were noted, with some parts of the basement glomerular membrane showing wrinkling and duplication under electron microscopy (Fig. 3). Approximately 20 days later, the sCr level improved to 1.05 mg/dL with an eGFR of 42.2 mL/min/1.73 m^2^, and the patient was discharged. The sCr level gradually improved, although her renal function improvement was limited, and the Cr level was 0.96 mg/dL and eGFR was 46.5 mL/min/1.73 m^2^ approximately at the 3 month follow-up.

**Table 1:** Laboratory clinical transition of three cases

Day	Case 1			Case 2		Case 3			Normal range	
	− 300	0	86	0	33	− 428	0	76		
Hematology										
WBC		12,600	8600	5300	6600	6100	5700	5200	3900–9700	/µL
RBC		451	426	500	534	507	464	450	430–567	10^4^/µL
Hb		14.2	12.9	14.8	15.9	17.7	16.5	15.7	13.4–17.1	g/dL
Plt		435	429	207	212		333	269	153–346	10^3^/µL
Biochemistry										
AST		26	21	31		22	17	17	5–37	IU/L
ALT		20	12	26		16	14		6–43	IU/L
T-Bil		0.5	0.5	0.9			0.9		0.4–1.2	mg/dL
γGTP		24		39		48	36		0–75	U/L
LDH		214	183	189	157		179	169	124–222	IU/L
ALP		217		66	66	58	83	65	38–113	U/L
BUN		42	13	11	13		10	9	9–21	mg/dL
Creatinine	0.65	2.32	0.96	0.87	0.92	0.81	1.05	1.13	0.60–1.00	mg/dL
eGFR	71.6	17.72	46.52	74.38	69.97	81.4	60.92	56.21		
Glucose		99	111	100	94	112	137	112	65–109	mg/dL
Creatine Kinase		95		1122	78		47	56	57–240	U/L
CKMB				6					0–15	U/L
HbA1c		4.8		5.6			4.4		4.6–6.2	%
Triglyceride		98		142		78	153		30–149	mg/dL
HDL-Cholesterol		73		41		61	55		40–70	mg/dL
LDL-Cholesterol		114		137		148	137		70–139	mg/dL
Total Protein		7.9	8.1	7.5	7.3		7.5	7.1	6.5–8.5	g/dL
Albumin		4.9	5.0	4.8	4.8		4.5		3.8–5.2	g/dL
Uric Acid		1.9	3.8	6.6		6.5	3.1	5.6	3.5–6.9	mg/dL
Na		130	139	140	139		139	138	135–145	mEq/L
K		2.5	4.0	3.9	4.4		4.1	4.5	3.5–5.0	mEq/L
Cl		109	102	105	103		106	106	96–107	mEq/L
Ca		9.4	10.4	9.4	9.4		9.5	9.2	8.5–10.2	mg/dL
P		3.3	3.8	2.7	3.9		1.6	2.7	2.0–4.5	mg/dL
Mg		2.5							1.8–2.6	mg/dL
Serology										
CRP		0.02	0.01	0.10	0.06		0.02		0.00–0.29	mg/dL
IgG		1098		1102			1341		870–1700	mg/dL
IgM		37		79			54		46–260	mg/dL
IgA		154		265			259		110–410	mg/dL
C3		116		129			105		86–160	mg/dL
C4		54		28			20		17–45	mg/dL
CH50		60.2		44.8			37.8		25–48	/mL
ANA		40		40			40		~ 40	
MPO-ANCA		< 1.0					< 1.0		~ 3.49	U/mL
PR3-ANCA		< 1.0					< 1.0		~ 3.49	U/mL
RF		5					5		~ 15	IU/mL
Anti-SS-A antibody		–							~ 0.9	
Anti-SS-B antibody		–							~ 0.9	
IEP		None					None			
Arterial blood gas										
pH		7.297								
pCO_2_		30.1								mmHg
pO_2_		113								mmHg
HCO_3_^−^		14.3	30.5	28.2	29.6		29.0	27.7		mmol/L
Anion gap		5.5								mmol/L
Urinalysis										
pH		5.0	7.5	6.0			6.0	5.5	5.0–8.0	
Protein		(2 +)	(−)	(−)		(−)	(−)	(−)		
Occult blood		(3 +)	(−)	(−)		(−)	(−)	(−)		
Glucose		(4 +)	(−)	(−)		(−)	(1 +)	(−)		
Osmic pressure		382							50–1300	mOsm/L
FEua		45.3							4.0–14.0	%
%TRP		61.7					73.8		60.0–90.0	%
TTKG		14.7								
Total protein		3.20								g/gCre
NAG		28.5	2.0	5.7			1.8	17.4		IU/L
β2MG		22,677	158	119			2533	389		µg/L
BJP		None		None			None			
Urine sediment										
WBC		1–4	~ 1	1–4			~ 1	1–4		/HPF
RBC		5–9	~ 1	~ 1			~ 1	~ 1		/HPF

*WBC* white blood cell, *RBC* red blood cell, *Hb* hemoglobin, *Plt* platelet, *CKMB* creatine kinase muscle/brain, *ANA* antinuclear antibody, *MPO* myeloperoxidase, *PR3* proteinase3, *ANCA* anti-neutrophil cytoplasmic antibody, *SS* sjögren’s syndrome, *IEP* immunoelectrophoresis, *FEua* fractional excretion of uric acid, *TRP* tubular reabsorption of phosphate, *TTKG* transtubular K gradient, *NAG* N-acetyl-β-D-glucosaminidase isoenzyme, *β2MG* β2-microglobulin, *BJP* bence jones protein, *HDL* high-density lipoprotein, *LDL* low-density lipoprotein, *eGFR* estimated glomerular filtration rate, *Ig* immunoglobulin, *AST* aspartate aminotransferase, *ALT* alanine aminotransferase, *T-Bil* total bilirubin, *γGTP* gamma-glutamyl transferase, *LDH* lactate dehydrogenase, *ALP* alkaline phosphatase, *BUN* blood urea nitrogen, *HbA1c* hemoglobin A1c, *CRP* C-reactive protein, *IgG* immunoglobulin G, *IgM* immunoglobulin M, *IgA* immunoglobulin A, *pCO₂* partial pressure of carbon dioxide, *pO₂* partial pressure of oxygen

### Case 2

A 48-year-old man with no relevant medical history was referred to our department for a decline in renal function. The patient had been taking Benikoji CholesteHelp^®^ for 2 years because of high LDL cholesterol level (130–140 mg/dL). He was also taking calcium, docosahexaenoic acid, and eicosapentaenoic acid supplements. The patient had no history of renal dysfunction, with a sCr level of 0.88 mg/mL and an eGFR of 74 mL/min/1.73 m^2^ 3 months prior. All supplements were discontinued approximately 1 month prior, following social media reports on kidney injury caused by Benikoji CholesteHelp^®^. Twenty-one days prior, his renal function worsened with a sCr level of 1.12 mg/dL and an eGFR of 56.76 mL/min/1.73 m^2^, and he was referred to our department. On his first visit, his blood pressure was normal. Although there was slight bradycardia with a pulse rate of 42 beats/min, electrocardiography revealed no abnormalities. Physical examination findings were normal, with no lower limb edema. The sCr level recovered to 0.87 mg/dL, and eGFR was 74.38 mL/min/1.73 m^2^, matching the level before renal dysfunction onset. The serum potassium (3.9 mEq/L) and phosphorus (2.7 mg/dL) levels were within the normal range. Urinary excretion of potassium or phosphorus was not elevated. Hematuria and proteinuria were not observed, and tubular injury markers were normal. Autoantibodies or M proteins were not detected. A renal biopsy was deemed unnecessary because renal dysfunction had improved on the day of the visit. The blood creatine kinase level was high, at 1122 IU/L, possibly due to the patient playing tennis 2 days before the first visit. It normalized to 78 IU/L at the follow-up 33 days later. The slight bradycardia was considered due to the patient’s regular exercise habits of playing tennis and running. At the 33-day follow-up, blood tests showed no abnormalities (Table 1).

### Case 3

A 47-year-old man with no relevant medical history was referred to our department for kidney dysfunction and suspected FS. On one-year prior medical examination, his sCr level and eGFR were 0.81 mg/dL and 81.4 mL/min/1.73 m^2^. The patient had been taking Benikoji CholesteHelp^®^ for 6 months and noticed foamy urine 1 month prior. Therefore, he stopped the supplement and visited the local doctor 10 days prior. Mild renal dysfunction was detected, with a sCr level of 1.09 mg/dL and an eGFR of 58.5 mL/min/1.73 m^2^. Urinary β2 microglobulin and N-acetyl-β-D-glucosaminidase (NAG) levels were high, at > 50,000 μg/L and 36.8 IU/L. The serum potassium level was 3.0 mEq/L, and the patient had glycosuria. He was instructed to consume potassium-rich foods and was referred to our department because of kidney dysfunction and suspected FS. The sCr level was 1.05 mg/dL, almost unchanged. Urinary β2 microglobulin and NAG levels were both reduced to 2598 μg/L and 1.8 IU/L. Hematuria and proteinuria were not observed. The serum potassium level was 4.1 mEq/L, which had already increased. The serum phosphorus and blood uric acid levels were low, at 1.6 mg/dL and 3.1 mg/dL, and the %TRP and FEUA levels were 73.8% and 23.7%, indicating that the excretion of phosphorus and uric acid levels remained high. These data suggested partial improvement of the tubulointerstitial injury; however, the tubular dysfunction persisted. Furthermore, the improvement in the serum potassium level was at least partially due to food intake. Autoantibody or M protein tests were negative. Although the sCr level showed no significant change during outpatient follow-up, the serum levels of potassium, phosphorus, and blood uric acid increased to 4.5 mEq/L, 2.7 mg/dL, and 5.6 mg/dL, respectively (Table 1). His electrolyte abnormalities improved, and metabolic acidosis was not observed; therefore, a renal biopsy was deemed unnecessary.

## Discussion

We report three cases where renal dysfunction developed in several months up to 2 years from the start of the administration of Benikoji CholesteHelp^®^. Case 1 had severe renal dysfunction and FS; thus, a renal biopsy was performed. Light microscopy revealed no active tubulointerstitial nephritis or glomerulonephritis, and the main pathological findings were interstitial fibrosis and tubular atrophy. On electron microscopy, no apparent morphological abnormalities were observed in the tubular epithelial cells. Ga scintigraphy findings, reflecting inflammatory cell infiltration in the interstitium [13], were also negative, and her renal function improved without immunosuppressive therapy after prompt supplement discontinuation on admission. Two other cases also had a favorable prognosis after supplement discontinuation alone.

Acquired FS is caused by diseases such as paraproteinemia and Sjögren’s syndrome [14, 15] or exposure to heavy metals and drugs. Drug-induced FS is caused by anti-human immunodeficiency virus drugs, anticancer drugs such as platinum compounds and alkylating agents, and antibiotics, including gentamicin and valproic acid [5]. Our patients had no history of any of these conditions. We suspected drug-induced nephropathy during admission and discontinued Benikoji CholesteHelp^®^. As FS subsequently improved without immunosuppressive therapy, supplements were the most likely cause. Simultaneously, a series of cases of renal dysfunction after taking Benikoji CholesteHelp^®^ with specific lot numbers was reported [7–11]. Although the total duration of supplementation in these reports varied from 6 to 18 months, the duration of supplementation of specific lot numbers and the periods between the exposure of puberulic acid and the disease may have been similar. Additionally, if there have been some differences in the periods and extent of onset, this may also be influenced by differences in the daily dose.

As the kidney and liver are the main excretory organs, they are frequently susceptible to damage caused by harmful drugs or supplements. In the kidney, the tubulointerstitium is the most common site of damage caused by these substances [16]. Mechanisms of drug- and supplement-induced kidney damage include direct damage dependent on drug dosage and duration of administration, allergic reactions, and other immune mechanisms, and indirect kidney damage caused by changes in renal blood or urine flow. The mechanism of kidney injury varies widely between drugs [4]. Many drugs that cause FS are taken up by the proximal tubule from the blood and induce tubular toxicity depending on the degree of this uptake [5]. For example, cisplatin is more easily taken up into the renal tubules via organic cation transporters than carboplatin; therefore, it is more toxic to tubules [5]. If the drug is discontinued early, the symptoms frequently improve within a few days to weeks. However, if long-term administration leads to tubular necrosis, it takes several months or more for the improvement, and sometimes the condition becomes irreversible [4].

The patients in all three cases showed rapid improvement after supplement discontinuation. In Case 1, the main renal biopsy findings were tubular atrophy and interstitial fibrosis, which do not deviate from the previously reported renal biopsy findings in FS [5, 17]. These were consistent with the negative result on Ga scintigraphy, which reflects inflammatory cell infiltration in the interstitium [13]. We suspected a direct effect on tubular epithelial cells in the present cases, rather than an allergic or immune mechanism, similar to that of the known FS-causing agents, for the following reasons, in addition to renal pathology findings. All three patients had no allergic symptoms, such as increased eosinophil count in the blood or skin rash. Additionally, renal damage improved after the supplement was discontinued, without the need for steroids or other immunosuppressive therapies.

Renal biopsy is frequently performed to evaluate the degree of tubular injury and interstitial fibrosis in patients with drug-induced FS. Although the histological findings are nonspecific, they may include swelling or vacuolization of tubular epithelial cells, tubular atrophy, and interstitial fibrosis to varying degrees. Electron microscopy may reveal swelling and abnormal mitochondrial morphology in proximal tubular epithelial cells [5, 17]. The renal tissue in Case 1 showed no glomeruli or blood vessel abnormalities. Only abnormalities in the renal tubules and interstitium were observed, which were not significantly different from those of the previous reports of FS [5, 17]. However, almost intact findings on electron microscopy may be due to the possibility of sampling a mildly damaged area. Conversely, the persistence of mild chronic kidney disease (CKD) at 3 months after drug discontinuation in Case 1 can be explained by the presence of interstitial fibrosis in approximately 40% of the area on light microscopy.

The possible mechanisms of renal function decline in drug-induced FS include tubular atrophy and interstitial fibrosis resulting from prolonged renal damage, dose-dependent tubular necrosis caused by drugs [18], decreased creatinine secretion due to tubular damage, and prerenal nephropathy stemming from polyuria [5]. Case 1 had FS and AKI but showed rapid recovery to a large extent after supplement discontinuation. Considering no diffuse tubular necrosis findings on renal biopsy, we suggest the possibility that the increase in sCr level in Case 1 was at least partly due to a temporary decrease in creatinine secretion [19].

Red yeast rice has organ-protecting and cholesterol-lowering effects; however, adverse events, such as acute liver damage and rhabdomyolysis, have also been reported [20, 21]. Song et al. reported that an overdose of the active ingredient lovastatin or contamination with the nephrotoxic citrinin and aflatoxins can cause these events [22]. The kind of red yeast rice bacteria used in Benikoji CholesteHelp^®^ has been found to be citrinin-free [23]. However, puberulic acid was accidentally contaminated in the supplements in these cases. Puberulic acid is known to have antimalarial effects and potent cytotoxic effects [12]; however, to date, its nephrotoxicity has not been investigated. In an experiment conducted by the Ministry of Health, Labour and Welfare of Japan (not yet peer-reviewed or published), renal necrosis was observed in rats administered puberulic acid. To date, this substance is considered the most likely cause of renal insufficiency.

In Case 1, telmisartan was discontinued during hospitalization. Regarding the possibility of FS caused by angiotensin II receptor blockers (ARBs), Juan et al. reported a case of FS and AKI caused by metamizole and gemfibrozil in a patient taking valsartan. Renal biopsy results indicated that the reduced glomerular blood flow caused by valsartan may have promoted tubular necrosis [24]. However, to date, no report has indicated that ARBs are primarily involved in the onset of FS or tubular necrosis. Therefore, the supplementation of Benikoji CholesteHelp^®^ is likely to be the primary cause.

All three cases had no other medical history or comorbidities that could be likely causes for FS and they received no other treatment, including immunosuppressive therapy. Furthermore, both renal dysfunction and FS appear to have improved with supplement discontinuation alone. Given that the onset of FS has been reported on supplementation with specific lot numbers of Benikoji CholesteHelp^®^ [25], the supplement ingredients were likely the cause. However, the current report has some limitations. First, even three cases are not sufficient and entail a possibility of bias, meaning that further evidence is required to accurately determine appropriate treatment and prognosis. Second, the electron microscopy findings from the renal biopsy in Case 1 showed no abnormal findings in the tubules on electron microscopy, despite the existence of tubular injury on light microscopy, suggesting the possibility that the sample was taken from an area with relatively mild damage. Third, three cases received different concomitant medications or supplements, which could partly affect the clinical cause, even though these were not the direct cause of renal insufficiency.

In conclusion, we report three cases of FS after administering Benikoji CholesteHelp^®^. Further research is needed to determine the causative agent and mechanism underlying renal damage. Nevertheless, the cases reported here are a valuable addition to growing evidence. This case series also suggests that CKD persists in some patients and is possibly attributable to the degree of tubular atrophy and interstitial fibrosis, although the clinical course after supplement discontinuation was generally favorable. In FS caused by supplements or drugs, early discontinuation of the causative agent is critical. Patients should be informed that supplements and medicines consumed for health purposes may cause adverse effects, including kidney dysfunction, and healthcare providers should enlighten patients about the potential risks of all products that they have consumed.
